# The influence of a verbal prompt on school lunch fruit consumption: a pilot study

**DOI:** 10.1186/1479-5868-4-6

**Published:** 2007-03-05

**Authors:** Marlene B Schwartz

**Affiliations:** 1 Rudd Center for Food Policy and Obesity, Yale University Department of Psychology, New Haven, CT 06520-8369, USA

## Abstract

**Background:**

This study evaluated an environmental intervention intended to increase consumption of the fruit serving among elementary school children participating in the National School Lunch Program (NSLP).

**Methods:**

Children's fruit consumption was measured in two schools by observation. In the intervention school, cafeteria workers provided the verbal prompt, "Would you like fruit or juice with your lunch?" as the children stood in line in front of the fruit serving options. The control school had the same fruit and 100% juice options available, but the cafeteria workers did not provide a verbal prompt to take a fruit serving. Two variables were assessed: (1) Did children leave the lunch line with a fruit serving on their trays? and (2) Did they subsequently eat the fruit serving?

**Results:**

The average percentage of children who took a fruit serving was 60% in the control school and 90% in the intervention school. In both schools, approximately 80% of children ate the fruit on their tray. As a result, nearly 70% of the children in the intervention school consumed a fruit serving at lunch, while fewer than 40% did so in the control school.

**Conclusion:**

A simple verbal prompt appears to have a significant impact on the likelihood that children will take, and subsequently consume, a fruit serving as part of their purchased school lunch. If these findings are replicated, policymakers may consider adding verbal prompts to the serving policy of the NSLP in an effort to increase fruit consumption among school children.

## Background

The National School Lunch Program (NSLP) was developed in 1947 to ensure that American schoolchildren had access to a nutritionally balanced and affordable lunch. When the NSLP was initially developed, malnutrition due to too few calories was the primary health concern. Paradoxically, six decades later many children are still suffering from malnutrition, although now the primary concern is with children consuming too many calories, and the accompanying increase in the rates of childhood obesity [1]. Children are eating more calories than they are expending, and the sources of those calories are inconsistent with recommended guidelines [2]. In particular, fruit and vegetable consumption is notably lower than recommended, with 78% of high school students failing to consume five or more daily servings of fruits and vegetables [3].

A number of educational strategies have been used to increase children's consumption of fruit and vegetables. For example, the 5 a Day for Better Health Program is a large national nutrition education initiative designed to teach about the benefits of eating fruits and vegetables [4]. Nutrition education alone, however, may not be enough to change how children eat. One study found that children know which foods are most healthful, but despite this knowledge, are more likely to choose foods based on convenience and taste [5].

A key determinant of intake is the availability of certain foods. In the school environment, food availability is regulated by the NSLP guidelines. Elementary school lunches must offer 5 components: a 2 oz meat/meat substitute, 8 oz milk, 1 serving of grain, and two servings (3/4 cup) of a fruit/vegetable, and. It is important to note that although all components must be available, children are only required to take three of them. This provides choice and flexibility, but also creates a situation where some children who buy lunch will not benefit from certain key nutrients. In fact, an evaluation of the nutritional quality of school lunches found that there was a notable difference between the quality of what was available in the cafeteria and the quality of what children actually chose to eat. In other words, many schools offered options that were consistent with nutritional standards, but the average lunch actually served to the children was not [6].

This leads to the question: How do we encourage children to choose more fruits and vegetables at school? A number of researchers have examined strategies such as price adjustment, convenience, promotion, and verbal prompts by cafeteria workers on the fruit and vegetable intake of children [7]. In one study, Perry and colleagues found that elementary school children consumed more fruits and vegetables in response to a cafeteria-based intervention that included presenting fruits and vegetables attractively, taste tests, maintaining variety, and verbal encouragement by the cafeteria workers to try the fruits and vegetables [8]. Because all of these components were included simultaneously, the influence of each component independently is not yet known.

The aim of the present study was to isolate and test the influence of having cafeteria staff provide a verbal prompt to the children to take a fruit serving when purchasing a school lunch. Specifically, this research study: (a) tested the hypothesis that a verbal prompt from the cafeteria workers would increase the number of children who took the fruit serving, and (b) compared the likelihood of fruit and juice consumption in the intervention versus control condition.

## Methods

### Schools

Two comparable elementary schools in the same school district in a small New England town were invited to participate in the present study and were randomly assigned to condition. Both schools have predominantly Caucasian students (89% intervention, 90% control), very few students who qualify for free or reduced lunch (fewer than 10% in each school), and very high rates of 4th graders scoring at or above grade level on the Connecticut Mastery Tests (91% for the intervention school, 87% for the control school). The enrollment is 309 students at the intervention school and 337 at the control school. Both schools participate in the same district-wide food service program, therefore, have the same foods available each day. The food service director reported that on average, 50% of children buy lunch at each of the two schools.

### Intervention

The idea for the intervention emerged from the school district's Health Advisory Committee and the author was asked to help the district assess the impact of the intervention. Prior to the beginning of the school year, the district superintendent, food service director, and school principals agreed to the protocol for the study and the protocol was approved by the Yale University Human Subjects Review Board.

The food service regularly offered children a choice of at least two types of fresh or canned fruit, and one or two types of 100% juice, each day. In the intervention school, the cafeteria workers were instructed by the food service director to provide the following verbal prompt to each child while he or she was standing front of the fruit servings: "Would you like fruit or juice?" The implication of this statement was that children were expected to take a fruit serving, however, if a child indicated that he or she did not want fruit or juice, the food service worker did not prompt further. In the control school, no changes were made; the same fruit and juice options were available each day in the cafeteria line, but no verbal prompt was given.

A few weeks into the school year, the food service director reported that the intervention had been successfully implemented. The author visited the intervention school and spoke with the cafeteria workers to verify this. The present study did not influence the fruit choices provided in the schools, as the cafeteria workers and food service providers did not know which days would be used for data collection.

### Observational data collection

The first observation day was in January 2005 and the second was in March 2005. The intervention and methods of observation on the two days were identical. The district superintendent and principals of each school gave permission for parent volunteers to observe fruit consumption in the cafeteria for two days. The volunteers were all parents of children in the school. They were instructed to casually observe what children were eating without initiating conversation with the children. If asked what they were doing, the observers were instructed to say that they were "just interested in seeing how the children were enjoying their lunch." During each lunch period, the observers recorded the following information for each child who purchased lunch: Did the child take fruit or juice? Did the child consume the fruit or juice? If the child consumed the fruit or juice, the observer rated whether it was partially (i.e., less than half) or fully consumed (i.e., equal or greater than half). There were four lunch periods in each school, corresponding to grades 1 through 4.

### Analyses

The data were first analyzed for the two days separately, and then averaged. Fewer than 3% of the observations indicated "partial consumption," so this variable was collapsed into two levels (i.e., yes or no) for the analyses. Frequency analyses were used to determine: (a) the number of students who purchased lunch in each school, (b) the percentage who took fruit and juice, and within that (c) the percentage who ate the fruit and drank the juice. To calculate the odds ratios for taking the fruit serving, and eating the fruit serving, the data were analyzed using the formula to calculate the odds ratios and confidence intervals in an unmatched case control study [9].

## Results

### Purchasing lunch and choosing fruit: day 1

The intervention school will be referred to as School A and the control school will be referred to as School B. On the first day of the intervention (Day 1) approximately half of the children in each school purchased the school lunch (School A: 48%; School B: 52%). The four lunch choices available at both schools were: Quesadilla (69%), Bagel yogurt plate (19%), Caesar salad (9%), or Soup and sandwich (3%). The fruit choices were: fresh apples, fresh oranges, and canned pears, and the 100% juice choices were apple and orange. At School A, 76% of those who purchased a school lunch took a piece of fruit and 21% took a carton of juice; 3 children explicitly stated that they did not want to take either. At School B, 45% of the children took a piece of fruit, 20% took juice, and 35% took neither.

Odds ratio calculations indicate that students at School A were nearly four times as likely to take fruit than students at School B (OR = 3.96, CI 2.2 – 7.0), but they were not more likely to take juice (OR = 1.0, CI 0.5 – 2.0). Once the food or drink was on the children's tray, they were more likely to eat or drink it than throw it away. Among the children who took fruit at School A and School B, 70% and 69% ate it, and among the children who took juice, 64% and 58% drank it. Overall, the likelihood of eating fruit among children in School A was three and half times that of children in School B (OR = 3.5, CI 2.0–6.2), but the likelihood of drinking juice was similar (OR = 1.1, CI 0.6 – 2.5).

### Purchasing lunch and choosing fruit: day 2

On the second day of data collection, similar numbers of children bought lunch at each school and the majority chose popcorn chicken (88%) or hot dog (11%). The fruit choices were apples, canned mandarin oranges, and grapes, and the juices were apple and orange. At School A, 48% of the children took fruit, 38% took juice, and 14% said they did not want either one. At School B, 32% of the children took fruit, 23% took juice, and 45% chose neither. Odds ratio calculations indicate that on Day 2, children at School A, children were nearly twice as likely to take fruit (OR = 1.9, CI 1.1 – 3.3) and juice (OR = 2.1, CI 1.2 – 3.8) than at School B.

**Figure 1:**
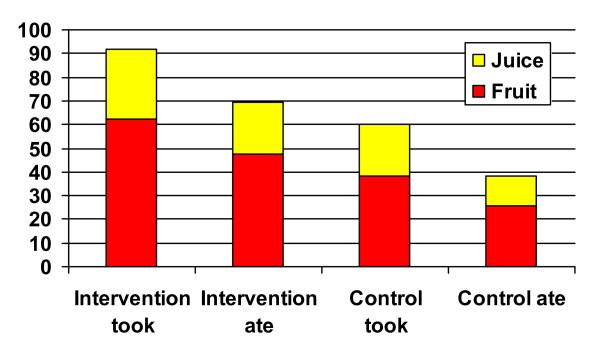
**Mean Percentage of Children Taking and Eating Fruit Servings**. Bars represent the percentage of children taking, and subsequently eating, fruit and juice at each school.

Again, once children at School A had the fruit or juice on their trays, they were quite likely to eat or drink it (87% and 88% respectively). Surprisingly, at School B, the children were somewhat less likely to eat or drink the fruit option they had chosen (65% and 62%). Overall, on Day 2 the odds ratios were that children at School A were more than twice as likely to eat fruit (OR = 2.3, CI = 1.3 – 4.2) or drink juice (OR = 2.9, CI 1.5 – 5.5) than children in School B. The average frequencies of children at each school taking and subsequently eating fruit and juice are presented in Figure 1.

## Discussion

At this time, many states are actively debating legislation to address childhood obesity and the Federal government has mandated that every district must have a Wellness Policy. The present study suggests that a relatively simple intervention – verbally prompting children to take the fruit option in their school lunch – may lead to a substantially greater intake of fruit. In these observations, when fruit and juice were simply made available, approximately 60% of children chose one or the other (38% took fruit and 22% took juice). This number increased to over 90% when children were prompted to take fruit or juice by a staff member (62% took fruit and 29% took juice).

The observations in the present study also suggest that most children consumed the fruit they took. The implication is that by increasing the number of children who sit down to eat with fruit on their trays, the number of children who eat fruit will also increase. Overall, close to 70% of the children at the intervention school consumed a fruit serving at lunch, whereas fewer than 40% of the children in the control school ate a fruit serving at lunch that day. If these findings are replicated in further studies, it suggests that a simple intervention such as the one carried out in the present study could significantly increase the number of servings of fruit that American children consume.

Some research has found that children who participate in the NSLP already have higher intake of fruits and vegetables than children who do not [10]. It is possible that this is because school lunches always offer a fruit/vegetable serving, whereas lunches brought from home do not. There is a significant amount of research to suggest that people are very likely to eat something once it is in front of them, and minor steps to increase how easy and convenient it is to get that food will significantly increase the likelihood it will be eaten [11]. Efforts to increase children's access to healthier foods and decrease access to unhealthful foods have the potential to promote positive dietary changes.

### Limitations

The present pilot study has a number of methodological limitations. First, only two schools participated in the study, and they were both high performing schools with very few students who qualify for a free or reduced lunch. In order to establish the generalizability of these findings, a larger sample that is representative of American public schools is needed. Second, the burden of the observation was reduced by having two observers at each site, but each observer assessed only half of the children, and interrater reliability was not tested. Obtaining adequate interrater reliability will be important in future research to reduce the risk of observer bias. Third, the present study used parent observers instead of researchers unfamiliar to the students. Using parents has the benefit of minimizing disruption and the risk of student awareness of being observed in the cafeteria, but it also has several drawbacks. Specifically, parents may not have been objective in their observations because they knew some of the children, and although efforts were made to keep the parent observers naive to the study hypotheses, the fact that they spend time in the school outside of this study created considerable risk of the parents figuring out the hypotheses. Future studies that employ similar methods would be strengthened by using a combination of familiar and unfamiliar adults for the data collection, taking stronger measures to ensure observers are naïve to the study hypotheses, and establishing interrater reliability.

There are also potential concerns that arise if more children take the fruit serving at lunch. One is that the absolute number of wasted servings might increase. In the present study, waste of fruit and juice at the intervention school were 15% and 7%, respectively; at the control school, the average waste of fruit was 13% and juice was 8%. These findings suggest that encouraging children to take the fruit, and having a greater number of children actually take the fruit, did not result in increased waste. However, this study would need to be replicated in order to come up with more definitive estimates.

Another important question is whether increasing fruit servings through this type of intervention will ultimately improve children's diets. It is possible that children will not compensate for the additional calories from the fruit or juice, which would undesirably increase caloric intake overall. A previous study on the ability to compensate for calories in liquid versus solid carbohydrates found that people are better at compensating for calories from solid versus liquid sources [12]. This suggests that children may be more likely to compensate adequately for the fruit, but the calories from the juice may to contribute to excess calories. An evaluation of the USDA Fruit and Vegetable Pilot Program found one school where foodservice staff reported a 25% reduction in doughnut and 50% reduction in dessert sales with the introduction of the pilot, suggesting students were compensating [13]. Future research in both naturalistic and lab settings should examine how this intervention impacts what children eat at that meal and subsequent meals to measure the net change in caloric intake and nutrients. Finally, increasing vegetable consumption may be more difficult than increasing fruit consumption, so future research could examine whether a verbal prompt is adequate in increasing vegetable consumption.

## Conclusion

While nutrition education is important, it is possible that the most efficient and effective way to improve children's nutrition is to change their food environment. The present study suggests that the simple intervention of a verbal prompt may have a significant impact on fruit consumption. In the school where children were prompted to take fruit as part of their school lunch, significantly more children were observed eating the fruit serving than in the control school, where the fruit was available, but there was no prompt. Changing the regulations of the NSLP so that cafeteria workers prompt the children to take the fruit may be an effective intervention to increase students' fruit consumption nationally.
